# Arthroscopic Management of Pigmented Villonodular Synovitis of the Hip: A Systematic Review

**DOI:** 10.3390/jcm13216446

**Published:** 2024-10-28

**Authors:** Riccardo Giai Via, Matteo Giachino, Ahmed Elzeiny, Gianvito Santarsiero, Alessandra Cipolla, Salvatore Pantè, Francesco Bosco, Kristijan Zoccola, Alessandro Massè, Alessandro Aprato

**Affiliations:** 1 Department of Orthopaedic Surgery, Centro Traumatologico Ortopedico (CTO), University of Turin, Via Gianfranco Zuretti, 29, 10126 Turin, Italy; riccardo.giaivia@unito.it (R.G.V.); matteo.giachino@unito.it (M.G.); gianvito.santarsiero@unito.it (G.S.); alessandra.cipolla@unito.it (A.C.); salvatore.pante@unito.it (S.P.); alessandro.masse@unito.it (A.M.); 2 Department of Orthopaedics and Traumatology, Faculty of Medicine, Kafr El Sheikh University, Kafr El Sheikh 33516, Egypt; elzeiny1890@gmail.com; 3 Department of Precision Medicine in Medical, Surgical and Critical Care (Me.Pre.C.C.), University of Palermo, 90133 Palermo, Italy; 4 Department of Orthopaedics and Traumatology, G.F. Ingrassia Hospital Unit, ASP 6, 90131 Palermo, Italy; 5 Department of Orthopaedics and Traumatology, Ospedale San Giovanni Bosco—ASL Città di Torino, 10154 Turin, Italy; kristijan.zoccola@aslcittaditorino.it; 6 Department of Pediatric Orthopaedic Surgery, Ospedale Infantile Regina Margherita, University of Turin, Piazza Polonia, 94, 10126 Turin, Italy; alessandro.aprato@unito.it

**Keywords:** Pigmented villonodular synovitis, arthroscopic hip surgery, clinical outcomes, recurrence rate, synovectomy

## Abstract

**Background/Objectives**: Pigmented villonodular synovitis (PVNS) is a benign proliferation of synovial tissue that can cause joint damage. The hip, although less commonly affected than the knee, presents a challenging diagnosis and treatment, with magnetic resonance imaging (MRI) as the gold standard for detection. Surgical excision, arthroscopic or open, is the main treatment approach, but there is no consensus on the best strategy for the hip. The aim of this systematic review is to evaluate the clinical outcomes, complications, and revision rates associated with arthroscopic hip surgery for PVNS. **Methods**: A systematic review was performed following the PRISMA guidelines. Relevant studies were identified by searching four databases: PubMed, Scopus, Embase, and Medline. Selected articles were evaluated according to the criteria of levels of evidence (LoE). For retrospective studies, the Coleman Methodology Score (mCMS) was used. This systematic review was registered with the International Prospective Register of Systematic Reviews. **Results:** Six studies satisfied the criteria; these involved 77 patients (48% male, 52% female) with a mean age of 26.4 years and a mean follow-up of 54.3 months. MRI and biopsy confirmed the diagnoses, and arthroscopic synovectomy was the primary treatment. Success rates ranged from 80% to 100%, with a recurrence rate of 7.8%, 1.3% requiring revision surgery, and eight (10.4%) patients in three studies reporting conversion to THA. Complications included mild effusions and residual synovitis. All patients who underwent a subsequent total hip arthroplasty were affected by advanced osteoarthritis. **Conclusions:** Our systematic review reveals that the use of hip arthroscopy in diagnosing and treating PVNS has shown satisfactory results without increasing the risk of recurrence or complications and can return patients to their former activity levels, provided their preoperative osteochondral status is good and there is early management of PVNS of the hip joint.

## 1. Introduction

Pigmented villonodular synovitis (PVNS) is a non-cancerous condition characterized by either localized or widespread overgrowth of the synovial tissue, potentially leading to damage in the affected areas [1,2,3]. Although PVNS rarely becomes malignant, it is associated with the formation of nodules and villous or villonodular lesions in the synovial tissue. These growths can be well defined and lobulated or appear as aggressive, diffuse lesions [1,3,4]. The characteristic pigmentation in these lesions is due to the accumulation of hemosiderin, particularly in intra-articular forms. Histologically, the lesions exhibit polymorphic features [1,2,3,4]. In the latest edition of the WHO Classification of Tumors (5th ed. 2020), PVNS is classified under tenosynovial giant cell tumors (TGCT), which primarily manifest as diffuse-type proliferation [5].

Although PVNS primarily affects the synovium, it can spread to other joint tissues, such as the bursa or tendons and extra-articular tissues. The weight-bearing joints, particularly the knee and hip, are the most affected [6,7,8]. The incidence is estimated at 30.3 per million people per year for the localized form and 8.4 per million people per year for the disseminated form, with a predilection for young adults aged 20 to 40 years, with some studies reporting a slight female predominance [6,8,9]. PVNS presents a clinical presentation that varies widely among patients depending on the location and size of the mass. Common signs and symptoms include pain, swelling, limitation of range of motion, and fatigue. The clinical diagnosis is challenging, as the signs are nonspecific [6,7,8,9,10].

X-ray imaging is usually the initial diagnostic tool, revealing soft tissue swelling or bony lesions, including single or multiple cysts with sharp-edged bone erosion [10,11]. However, radiographs may appear normal in the early stages of the disease. MRI offers high sensitivity, revealing joint effusion, synovial expansion, and hemosiderin artifacts. [10,11] It represents the gold standard for diagnosis, preoperative planning, and postoperative evaluation. CT can be useful in biopsy-guided procedures for obtaining a definitive diagnosis [6,10,11].

Traditionally, surgical excision with synovectomy, through arthroscopic or open approaches, has been the main treatment [12,13,14]. The literature on arthroscopic treatment of PVNS of the hip is limited, with no consensus on the optimal treatment strategy [12,13,14,15,16]. The recurrent nature of the lesion, particularly in the knee, has stimulated the exploration of alternative treatments such as perioperative radiotherapy, intra-articular radioisotope injections, and chemotherapy with tyrosine kinase inhibitors [6,12,13,14,15,16].

The main objective of this systematic review is to analyze and synthesize the available evidence on clinical outcomes, complications, and revision rates associated with arthroscopic hip surgery for PVNS. Given the rarity of PVNS, especially in the hip joint, there is a need for comprehensive evaluation of the efficacy of arthroscopic intervention, which remains an evolving treatment modality. This review aims to determine whether arthroscopic surgery can offer long-term symptom relief, functional improvement, and reduced recurrence rates, compared with other treatment approaches. Moreover, the study seeks to explore the complications that may arise from this minimally invasive procedure, including potential risk of incomplete resection, joint instability, or damage to surrounding tissues. Additionally, the review will assess the necessity and frequency of revision surgeries, providing insights into the durability and sustainability of arthroscopic treatment for PVNS in the hip.

## 2. Materials and Methods

This research followed the guidelines outlined in the Preferred Reporting Items for Systematic Reviews and Meta-Analyses (PRISMA) [17]. To ensure accuracy, the literature search and study evaluation were conducted independently by three authors (RGV, AE, and GS). Any potential uncertainties were resolved by consulting a fourth author (MG).

### 2.1. Inclusion and Exclusion Criteria

The inclusion criteria for this systematic review focused on studies involving patients diagnosed with PVNS of the hip who were treated with arthroscopic surgery. To ensure a comprehensive and relevant analysis, only articles that met the following criteria were included: studies published in English, involving human subjects, with publication dates ranging from 2004 to April 2024. Additionally, a minimum mean follow-up duration of 32 months was required to evaluate the long-term outcomes of arthroscopic intervention. Only studies with levels of evidence (LoE) [18] between 1 and 4 were considered, including randomized controlled trials (RCTs), prospective studies, and retrospective studies, as these offer a balance between high-quality evidence and the availability of data in the context of a rare pathology such as PVNS.

The exclusion criteria were designed to maintain the scientific rigor and relevance of the included studies. Biochemical and in vitro research, case reports, editorials, book chapters, technical reports, preclinical studies, and review articles were excluded, as these do not provide direct clinical evidence on the management of PVNS in humans. Furthermore, studies with LoE 5, which are typically based on expert opinion or anecdotal evidence, were omitted to enhance the overall quality and reliability of the review’s findings. By focusing on higher levels of evidence and excluding studies with limited clinical applicability, this systematic review aimed to synthesize the most robust data available for assessing the outcomes of arthroscopic treatment of PVNS in the hip joint.

### 2.2. Search Strategy and Study Screening

**Figure 1:**
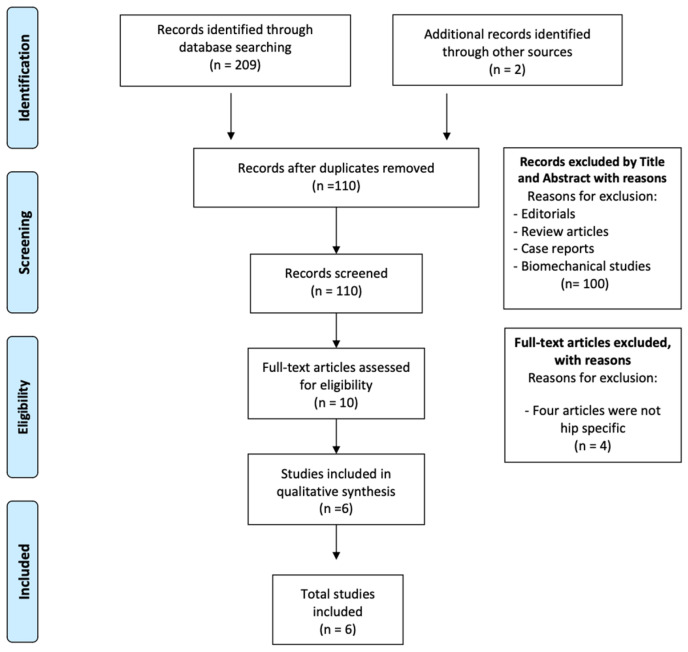
PRISMA flow diagram.

A comprehensive and systematic literature search was conducted in five databases (PubMed, Scopus, Embase, Medline, and Cochrane) using the following MeSH terms: ((villonodular synovitis) OR (pigmented villonodular synovitis)) AND ((arthroscop*) OR (endoscop*)) AND (hip). The search included studies published from 2004 to April 2024. Articles prior to 2004 were excluded to ensure a more modern and up-to-date literature base for the systematic review incorporating the most recent research. Furthermore, the exclusion reflects advances in arthroscopic hip techniques in recent years. After removing duplicates, 110 studies were included. After a review of the title and abstract of these studies, 100 studies were excluded, resulting in 10 eligible studies. After full-text evaluation, six studies met the eligibility criteria for qualitative analysis. A quantitative analysis was not possible due to a different representation of the data, which did not allow an adequate statistical comparison. The included studies directly reported functional outcomes, symptomatic period, and concomitant pathologies of the hip, as well as success, recurrence, and complication rates and conversion rates to total hip arthroplasty (THA) of patients undergoing arthroscopy for villonodular synovitis of the hip. The PRISMA diagram is shown in Figure 1.

### 2.3. Methodological Quality Assessment

**Figure 2:**
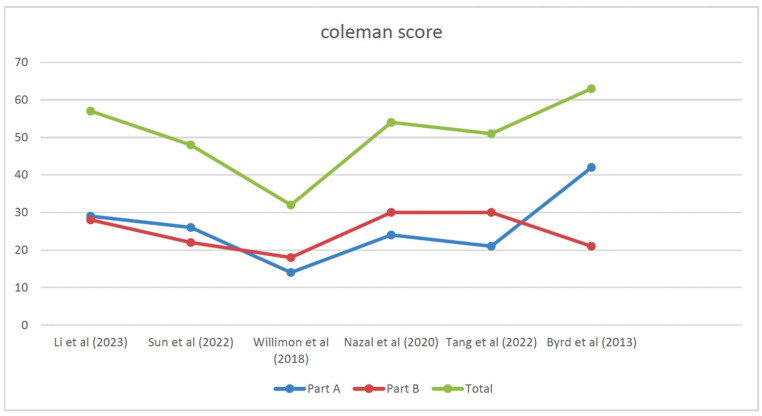
The Coleman Methodology Score (mCMS) [22,23,24,25,26,27], modified by Ramponi et al. [19].

Each selected article was evaluated according to the 2011 Oxford Centre for Evidence-Based Medicine levels of evidence (LoE), ranging from 1 to 5 [2]. Retrospective studies were evaluated using the Coleman Methodology Score (mCMS), modified by Ramponi et al. [19,20]. Two authors (RGV, AE) used this tool, while a third author (SP) was consulted to resolve any uncertainties. All contributors played a significant role in the ideation and design of the study, data collection, preparation of the manuscript, and completion of the final text changes. All authors approved the final version of the article. The systematic review was registered in the International Prospective Register of Systematic Reviews (PROSPERO), CRD42024540842, in May 2024 [21]. The Coleman score diagram is shown in Figure 2.

### 2.4. Data Extraction

Data extracted from the included articles were systematically recorded in Excel spreadsheets by two authors (RGV and AE), who worked independently before combining their data. This process included various details, such as author and year of publication, study design, patient sample size, mean age, mean BMI, duration of symptoms, mean follow-up time, patient positioning, duration of operation, concomitant diseases, success rates, complication rates, recurrence rates, rates of conversion to THA, postoperative protocols, subjective pre- and postoperative scores such as the Modified Harris Hip Score (mHHS), Harris Hip Score (HHS), Visual Analog Scale (VAS), Hip Outcome Score (HOS), Non-Arthritic Hip Score (NAHS), International Hip Outcome Tool (iHOT), and Low Extremity Functional Scale (LEFS).

### 2.5. Statistical Analysis

Statistical analysis employed R software (version 4.1.3, released in 202, R Core Team, Vienna, Austria). Descriptive statistical techniques were applied to the data collected from the included studies. Continuous variables were summarized using mean values, while measures of variability, such as standard deviation (SD) and range (minimum–maximum), were also utilized. Categorical variables were analyzed by determining absolute numbers and frequency distributions.

## 3. Results

Six studies met the inclusion criteria, underwent critical analysis and quality assessment, and were included in the systematic review. These studies were published between 2013 and 2023 and were classified as level IV, indicating the absence of comparative studies [22,23,24,25,26,27].

All studies included in this review were retrospective, except the study of Byrd et al., which was prospective [27]. Among the 77 patients in this review, 37 (48%) were male and 40 (52%) were female, with complete data availability. The mean age of the patients was 26.4 years (range, 4 to 66 years), and the mean follow-up period was 54.3 months, ranging from 3 to 120 months after treatment.

Three studies specified the affected side; in those studies, 17 patients had PVNS on the left hip and 22 on the right one [22,24,25]. Five studies reported the mean of time from symptoms till surgery, with a mean of 14.8 months (range 0.1–108 months) [23,24,25,26,27]. Three studies reported body mass index (BMI), which ranged from 16.2 to 31.7 kg/m^2^ [22,23,25]. All study and patient characteristics are summarized in Table 1.

### 3.1. Diagnosis

The diagnosis of PVNS in the included studies was based on various methods, including history, physical examination, and radiographs. Also, diagnosis might have been suspected on MRI, and it sometimes likely required diagnostic arthroscopy with biopsy confirmation to make a definitive diagnosis. Some patients were initially misdiagnosed with conditions such as synovitis, rheumatoid arthritis, avascular necrosis of the femoral head, synovial chondromatosis, femoroacetabular impingement (FAI), stress fracture of the femoral neck, hemorrhagic disorder, or hip effusion [23,24,26]. In four studies, MRI confirmed the diagnosis in 21 of 32 patients (65.5%) [23,24,25,26,27]. The types of PVNS observed were diffuse in 26 patients, nodular in 24 patients, and combined in seven patients [23,24,25,26,27]. Also, the femoral head and acetabular cartilage injuries were graded according to Tönnis grading in two studies [23,25]. In addition, concomitant diseases were identified in five studies (Table 2) [22,23,25,26,27].

### 3.2. Indication of Arthroscopy

Arthroscopic surgery was undertaken in cases where diagnostic ambiguity persisted regarding PVNS, particularly when joint integrity was largely preserved. The aim was to obtain biopsy specimens and relieve the symptoms of PVNS by performing an arthroscopic synovectomy without leaving substantial arthritic pain after treatment.

### 3.3. Surgical Technique

The primary treatment for PVNS of the hip joint involves focal or diffuse arthroscopic synovectomy, along with the treatment of accompanying conditions. Treatment descriptions scored a maximum of 10 points on the Coleman score in three studies [22,25,26]. The position of the patients was mentioned to be supine in four studies [22,25,26,27]. Mean operative times were reported in two studies as 103.1 ± 39 min and 96 ± 29.95 min, respectively [22,25]. Blood loss was mentioned in only one study, totaling 29.4 ± 28.6 mL [1]. Postoperative radiotherapy was mentioned in two studies involving 13 patients (Table 3) [23,26].

### 3.4. Postoperative Rehabilitation

Postoperative therapy after surgical management is shown in Table 3. Only two studies mentioned detailed rehabilitation therapy and a weight-bearing protocol in the postoperative period [25,26].

### 3.5. Final Reported Outcomes

Patient-reported subjective outcome scores were used in five studies, including the Modified Harris Hip Score (mHHS), the Visual Analog Scale (VAS), the Non-Arthritic Hip Score (NAHS), the International Hip Outcome Tool-12 (iHOT-12), the Hip Outcome Score Sport-Specific Subscale (HOS-SSS), the Hip Outcome Score Activities of Daily Living (HOS ADL), and the Lower Extremity Functional Scale (LEFS) [22,23,25,26,27]. Willimon et al. used the return to activities and MRI results of PVNS to assess outcomes [24]. The arthroscopic treatment of hip joint PVNS success rates were considered an improvement to patients’ functional outcomes, with a low rate of local recurrence either clinically or radiologically. The success rate ranged from 80% to 100% in the included studies (Table 4).

### 3.6. Complications

The recurrence rate was 7.8% (n = 6 of 77 hips), with only one hip (1.3%) requiring arthroscopic revision surgery after 65 months. However, the rest of the patients converted to THA. Eight patients (10.4%) in three studies reported conversion to THA within 3.7–6 years [22,23,27]. Other complications were reported among the selected studies. Byrd et al. reported a case of one patient suffering from residual synovitis and three patients having mild effusions [27]. Willimon et al. mentioned in their study a patient with residual osteoarthritis, although not converted to THA [24]. (Table 4).

## 4. Discussion

The most important finding of this systematic review is that arthroscopic hip surgery has been shown to be an effective surgical approach to treating patients with pigmented villonodular synovitis (PVNS) of the hip without increasing the risk of recurrence or complications.

PVNS is a rare pathology, with only a few studies in the literature regarding PVNS of the hip. The majority of published articles focus on the knee joint as it is the most frequent location of PVNS and report that a combined approach of anterior arthroscopy and posterior open approach has shown the best results with the lowest recurrence rate (rate between 9% and 25% of recurrence with a combined approach in diffuse PVNS compared with a rate of 92%–94% when an arthroscopic synovectomy or debridement was performed alone) [28,29]. To the authors’ knowledge, this is the only systematic review with articles dealing exclusively with patients treated arthroscopically at the hip for PVNS without including other anatomical regions that could reduce the specificity of the study. The best approach to performing a complete synovectomy, whether open or arthroscopic, for the hip joint remains poorly defined.

Due to the young age that characterizes patients affected by this pathology (in our review the mean is 26.4 years, ranging from 4 to 66 years), the joint preserving treatment seems to be the most reasonable choice [22]. Between conservative options, all authors consider arthroscopic synovectomy the first-line treatment because of its less invasive nature, lower morbidity rates, minor residual joint stiffness, and associated pain. In addition, the chance to obtain a biopsy gives the arthroscopic technique a dual diagnostic and therapeutic advantage [28].

As most authors reported, the first important point to underline is the importance of early diagnosis [24,30]. The aggressive nature of the disease can lead to rapid bone erosion and advanced osteoarthritis, which would make conservative treatment impracticable. In our review, the average time to diagnosis is still longer than one year (14 months). This is lower compared with other older studies [26,28], where the average delay to diagnosis varied between 13.5 and 25 months. This improvement can be explained by the growing accessibility to MRI, which plays an essential role in the early diagnosis of the initial stages of the pathology [31].

This diagnostic delay is attributed to lack of awareness of the pathology and to an incorrect differential diagnosis, with other diseases characterized by hip pain at a young age, such as rheumatology disorders, childhood septic arthritis, and juvenile idiopathic arthritis [24]. In more adult patients, the cause of delay can include an incorrect biopsy execution and an incomplete arthroscopic visualization, which can lead to false negatives. Initially, the major concern about using arthroscopy was the risk of getting incomplete access to the synovium compared with open surgery and, therefore, being unable to perform a radical synovectomy, which is essential to reduce the risk of recurrence [26]. Most studies localize the pathology in the peripheral compartment, which is challenging to visualize and, if not correctly investigated, can hide the lesions and delay the diagnosis. Delayed diagnosis leads to a degenerative impairment of the cartilage injuries until joint destruction, a condition that contraindicates the use of hip joint-preserving surgery. In this systematic review, the recurrence rate was 7.8% with a mean follow-up of 54.3 months. However, we did not find any study that directly compared the open and arthroscopic approaches for recurrence rate and hip joint preservation.

In all studies, associated lesions such as labral tears and FAI were found, so arthroscopy also plays a useful role in treating concomitant injuries by performing a synovectomy plus a labral repair and/or acetabuloplasty and femoral osteoplasty.

As we have said, most pathological lesions were found in the medial recess of the peripheral compartment [24]. For this reason, obsessive attention is required to clearly visualize both central and peripheral compartments with a 70° arthroscope and flexible instruments. Anterior and anterolateral portals were the most widely used in the included studies. Some authors suggested performing an extended capsulotomy to gain a complete view and using multiple portals to have dynamic access at the joint. Evaluating the most medial and lateral aspects of the peripheral compartment is mandatory to ensure the complete eradication of lesions and reduce the rate of recurrence [32,33]. Byrd et al. highlighted the importance of good peripheral vision, as the use of arthroscopy restricted only to the central compartment led to the failure of the diagnosis in two patients [27]. Tang et al. used the peripheral compartment first technique for all patients to gain an excellent view of the peripheral compartment while the tension of the hip capsule is maintained [26]. Additionally, the lateral decubitus position in the treatment of pigmented villonodular synovitis of the hip can be preferred, as it allows for easier access to the fovea. This improved access facilitates working within the joint space more effectively, enabling surgeons to perform a more radical synovectomy.

Several studies reported that the outcomes of open excisions increase the risk of osteoarthritic changes caused by the surgical invasiveness of the technique. With extensive hip involvement especially, this can represent a challenge in terms of surgical treatment [27,29]. The exposure of the hip joint by surgical dislocation can be achieved by several approaches, anterior or posterior. Undoubtedly, the surgical dislocation according to the technique described by Ganz, which uses an approach described as ‘trochanterotomy’ or ‘trochanteric flip’, allows the best exposure of the entire joint, femoral head, neck, and depths of the acetabular cup, while preserving the vascularization of the femoral head [34]. Ganz published his experience with this technique in 213 patients, some of whom had PVNS without avascular necrosis of the femoral head. The main risks identified in these 213 cases included neurapraxia (two cases, 1%), a loosening of the trochanter (three cases, 1.5%), and some heterotopic ossification (79 cases, 37%, including 68 cases of class 1, according to Brooker) [35]. Moreover, PVNS itself can lead to avascular necrosis, even if this is rarely reported in the literature [36]. Levy et al. mentioned that no matter which surgery is to be performed, surgeons must counsel patients about the high revision rate. One in four patients ultimately undergoes a second surgery, which may be required within 6 or 7 years after synovectomy without arthroplasty. Further development and innovation in hip arthroscopy may transform the treatment of PVNS [37].

No cases required conversion to open surgery to achieve a better exposition while performing hip arthroscopy. One study compared arthroscopic synovectomy alone with an open synovectomy combined with total hip arthroplasty (THA). This study found similarly good functional outcomes and no difference in recurrence after arthroscopic synovectomy and after synovectomy plus arthroplasty, but a higher rate of complications in the THA group. Repeating an arthroscopy in case of recurrence remains a less invasive and low-risk option than an arthroplasty, especially if THA is the primary treatment in young patients who will plausibly require revision surgery. Cheok et al. in their review concluded that hip arthroplasty had low rates of PVNS recurrence compared with synovectomy; however, it was associated with significant risk of aseptic loosening in the longer-term follow-up [38]. The combination of synovectomy associated with arthroplasty should be reserved only for patients with secondary advanced osteoarthritis. Secondary arthritis is the most frequent cause of reoperations with conversion to hip replacement [22].

Two studies mentioned postoperative radiotherapy in 13 patients, demonstrating recurrence rates slightly lower than those associated with surgery alone; however, they did not clarify the type of adjuvant therapies [23,24]. However, arthroscopic and/or open surgery remains the mainstay of treatment for PVNS for most patients. While radiosynoviorthesis with pexidartinib has been used for recalcitrant disease, recent understanding of the colony-stimulating factor 1 receptor (CSF1R) pathway and its paracrine and autocrine role in PVNS has led to the development of targeted inhibitors [39].

In the literature, the discovery of new antibody targeting medical therapies such as imatinib or etanercept has demonstrated encouraging responses with less toxicity compared with radiotherapy. However, no study included in this review mentioned the use of this innovative treatment [40,41].

## 5. Limitations and Future Directions

This systematic review presents several limitations that warrant attention. Firstly, the inclusion of low-quality studies, many of which are retrospective case series lacking comparison groups, impacts the overall strength of the evidence. Only one of the included studies was prospective, limiting the ability to draw robust conclusions. Secondly, the rarity of PVNS, particularly in the hip, has led to the inclusion of studies with a small number of patients, which limits the generalizability of the findings. This rarity also contributes to the heterogeneity of the available data, making it difficult to establish clear treatment protocols. Thirdly, the duration of follow-up across the studies varied significantly, ranging from 3 to 120 months. Such variability introduces potential bias and diminishes the reliability of conclusions regarding the long-term outcomes of arthroscopic treatment for PVNS. A more standardized and uniform clinical and radiological follow-up regimen would significantly enhance the validity and comparability of the data, allowing for a more accurate assessment of the procedure’s efficacy and recurrence rates.

Given these limitations, further high-quality research is essential to assess the long-term efficacy, safety, and cost-effectiveness of arthroscopic management for PVNS of the hip. Large-scale, prospective, randomized controlled trials with carefully matched control groups would provide more definitive evidence regarding the advantages of arthroscopic surgery over other treatment modalities. These studies should also aim to refine surgical techniques, optimize postoperative outcomes, and assess the role of adjunctive therapies in reducing recurrence rates.

Despite these limitations, this review highlights the advantages of arthroscopy in treating hip PVNS. It offers a high success rate, few complications, and a relatively low recurrence rate, while also allowing for the management of concomitant joint injuries. Moreover, arthroscopy, a minimally invasive procedure, preserves the integrity of the joint tissues more effectively than open synovectomy, which is particularly beneficial for patients who experience disease persistence or recurrence. In such cases, arthroscopy leaves the joint in a more favorable condition for future interventions, including potential arthroplasty, which may be required due to joint degeneration.

## 6. Conclusions

Hip arthroscopy has proven to be a reliable and effective approach for diagnosing and treating PVNS, demonstrating high success rates in symptom resolution and functional recovery. Importantly, this approach does not appear to increase the risk of recurrence or introduce significant postoperative complications when performed in patients with favorable preoperative osteochondral conditions. This minimally invasive technique allows many patients to return to their previous physical activity levels, which is particularly significant in the context of young and active individuals commonly affected by PVNS. Moreover, early detection and intervention through arthroscopic synovectomy can preserve joint integrity and function, potentially delaying or even preventing the progression to osteoarthritis and the need for total hip arthroplasty.

## Figures and Tables

**Table 1:** Main demographic characteristics of patients collected in studies included in the systematic review.

Authors (year)	Study Design (LoE)	No. of Patients (N)	Age (years), Mean ± SD (Range)	Male/Female (N/N)	Left/Right (N/N)	Type (Diffuse/Nodular/Combined) (N/N)	BMI (kg/m^2^), Mean ± SD (Range)	FU (mns) Mean ± SD (Range)	Symptomatic Period (mns), Mean ± SD (Range)
Li et al. (2023) [22]	Retrospective (IV)	20	34.2 ± 10.2	10/10	8/12	/	21.7 ± 2.1 (18.67–25.95)	50.3 ± 22.46	/
Sun et al. (2022) [23]	Retrospective (IV)	16	29.5 (16–66)	8/8	/	12/4/0	21.8 (16.2–27.3)	44.8 ± 38.2 (3–110)	16.9 (1–60)
Willimon et al. (2018) [24]	Retrospective (IV)	5	11 (4–17)	2/3	3/2	1/4/0	/	32 (12–63)	8.2 (0.1–31.1)
Nazal et al. (2020) [25]	Retrospective (IV)	14	32.69 ±12.7 (16–55)	6/8	6/8	5/9/0	27.8 ± 3.46 (21.8–31.7)	79.9 ± 22.4	21.33 ± 29.2 (2–108)
Tang et al. (2022) [26]	Retrospective (IV)	9	24.3 ± 11.2 (14–44)	2/7	/	5/4/0	/	55.8 ± 26.1 (24–84)	25.2 ± 24.4 (0.5- 60)
Byrd et al. (2013) [27]	Prospective (IV)	13	26.8 (14–46)	9/4	/	3/3/7	/	63 (24–120)	17 (2–60)

BMI: body mass index; /: not reported; FU: follow up; LoE: level of evidence; mns: months; SD: standard deviation; N: number of patients.

**Table 2:** Joint characteristics of patients with PVNS of studies included in the systematic review.

Authors (year)	Misdiagnosis (N)	MRI Diagnosis N (%)	Tönnis Grading (N, %)	Concomitant Pathology (N, %)
Li et al. (2023) [22]	/	/	/	Femoral head AVN (2, 10%), OA (3, 15%)
Sun et al. (2022) [23]	Inflammation (1), rheumatoid arthritis (1), femoral head AVN (1), synovial chondromatosis (2).	/	Head cartilage injury: Degree IV (5), degree III (4), degree II (4), and degree I (3). Acetabular cartilage injury: degree IV (5), degree III (1), degree II (1).	Labral tears (3, 18.8%), FAI (4, 25%).
Willimon et al. (2018) [24]	Transient synovitis (2), FAI (1), femoral neck stress fracture (1), bleeding disorder (1).	4 (80%)	/	/
Nazal et al. (2020) [25]	/	7 (50%)	Grade 1: (8, 57.1%), grade 2: (4, 28.6%), grade 3: (1, 7.1%).	Labral tears (5, 35.7%), FAI (10, 71.4%).
Tang et al. (2022) [26]	Groin or hip pain (7), coxa valga (1), hip effusion (1).	4 (44.4%)	/	Decreased internal rotation (< 15°) (6, 66.7%), FAI (6, 66.7%).
Byrd et al. (2013) [27]	/	6 (46%)	/	Cartilage lesions (7, 53.8%), labral tears (6, 46%), FAI (4, 30.8%).

MRI: magnetic resonance imaging; AVN: avascular necrosis; FAI: femoroacetabular impingement; OA: osteoarthritis; /: not reported; N: number of evaluation cases.

**Table 3:** Surgical position, surgical technique, and postoperative therapy of patients following endoscopic treatment of PNNS.

Authors (year)	Position	Operation Time (min), Mean ± SD (Range)	Surgical Technique (N)	Adjuvant Therapy (N)	Postoperative Protocol
Li et al. (2023) [22]	Supine	103.1 ± 39	Focal or diffuse arthroscopic synovectomy (detailed)	(0)	/
Sun et al. (2022) [23]	/	/	Arthroscopic synovectomy (name only) + labral repair (3) + Femoral osteoplasty (4) + Acetabuloplasty (2) + labral debridement (2)	PO radiotherapy (8)	/
				Diffuse (6)–Localized (2)	
Willimon et al. (2018) [24]	/	/	Arthroscopic synovectomy (name only)	/	/
Nazal et al. (2020) [25]	Supine	96 ± 29.95 (60–144)	Focal or diffuse arthroscopic synovectomy + concomitant pathology (detailed)	/	WB with crutches for 6 weeks
					After 6 weeks, one then no crutches + start rehabilitation
Tang et al. (2022) [26]	Supine	/	Subtotal synovectomy (detailed)	Radiosynoviorthesis (5)	Partial WB for the first 2 weeks and then full WB allowed
					Continuous passive and active ROM exercises recommended for first 4–6 weeks to prevent adhesions
Byrd et al. (2013) [27]	Supine	/	Excision diseased synovium + concomitant pathology (not detailed)	/	/

WB: weight-bearing; ROM: range of motion; /: not reported; N: number of evaluation cases; min: minutes; SD: standard deviation; PO: postoperative.

**Table 4:** Summary of postoperative outcomes, complications, recurrences, and revisions following endoscopic treatment of PVNS.

Authors (year)	Pre-Op Outcome Measures, Mean ± SD (Range)	Post-Op Outcome Measures, Mean ± SD (Range)	Success Rate (N/N, %)	Recurrence (N, %)	Revision (N)	Complication (N)	THA Conversion
Li et al. (2023) [22]	VAS 4.05 ± 0.94 HHS 45.30 ± 11.08 (25–64)	VAS 1.35 ± 1.79 HHS 71.6± 19.78 (46–95)	16/20 (80%)	4 (20%)	0	0	4
Sun et al. (2022) [23]	HOS ADL 63.1 ± 19.1(32–98.3) mHHS 54.8 ± 20.1 (10–77) iHOT-12 50.9 ± 15.4 (31–76.6) NAHS 51.6 ± 15.9 (20–84.4) VAS 6 ± 1.4 (4–8)	HOS ADL 79.7 ± 10.8 (58–97.6) mHHS 78.6 ± 9.1 (55.0–87) iHOT-12 74.7 ± 9.7 (55.6–91) NAHS 78.9 ± 18.7 (20.0–92.5) VAS 3.1 ± 1.2 (2–6)	13/16 (81%)	0	0	OA, 3	3
Willimon et al. (2018) [24]	/	Return to activities (4) MRI free of PVNS (3)	4/5 (80%)	0	0	OA, 1	0
Nazal et al. (2020) [25]	VAS 8.1 ± 1.1	VAS 3.2 ± 1.8 mHHS 74.1± 16 NAHS 78.9 ± 20 LEFS 64.9 ±17.9 HOS ADL 57.5 ± 12.5 HOS-SSS 73.7 ± 29.2 iHOT-33 67.9 ± 27.4	14/14 100%	1/14 (7.14%)	1, Arthroscopic	0	0
Tang et al. (2022) [26]	/	mHHS 94.6 ± 4.9 (84.7–100) iHOT-12 93.3 ± 20.2 (50–120)	9/9 100%	0	0	0	0
Byrd et al. (2013) [27]	mHHS 62 ± 17	mHHS 89 ± 17 mHHS improvement 27 (3–56)	12/13 (92%)	1/13 (7.69%)	0	Residual synovitis, 1; mild effusion, 3	1

THA: Total Hip Arthroplasty; mHHS: Modified Harris Hip Score; VAS: Visual Analog Scale; NAHS: Non-Arthritic Hip Score; iHOT-12: International Hip Outcome Tool; HOS-SSS: Hip Outcome Score Sport-Specific Subscale; HOS ADL: Hip Outcome Score Activities of Daily Living; LEFS: Lower Extremity Functional Score; /: not reported.

## Data Availability

The data presented in this study are available in the article.
